# The natural course of incidental ureteral polyp during ureteroscopic surgery: KSER research

**DOI:** 10.1186/s12894-023-01249-y

**Published:** 2023-06-03

**Authors:** Sung Yong Cho, Kyung-Jin Oh, Wonho Jung, Hyung Joon Kim, Sang Hyub Lee, Joo Yong Lee, Dong Sup Lee

**Affiliations:** 1 Department of Urology, Seoul National University Hospital, Seoul National University, College of Medicine, Seoul, South Korea; 2 Department of Urology, Chonnam National University Medical School, Gwangju, South Korea; 3 Department of Urology, Dongsan Medical Center, Keimyung University School of Medicine, Daegu, South Korea; 4 Department of Urology, Konyang University Hospital, Daejeon, South Korea; 5 Department of Urology, School of Medicine, Kyung Hee University, Kyung Hee University Medical Center, Seoul, South Korea; 6 Department of Urology, Urological Science Institute, Severance Hospital, Yonsei University College of Medicine, Seoul, South Korea; 7 Department of Urology, St. Vincent’s hospital, The Catholic University of Korea, Suwon, South Korea

**Keywords:** Ureteroscopy, Hydronephrosis, Urolithiasis, Polyps, Ureteral obstruction

## Abstract

**Background:**

The natural course of polypoid lesions in the ureter during ureteroscopic stone surgery was not yet clarified.

**Methods:**

Patient data were collected prospectively from six teaching hospitals between 2019 and 2021. Patients with polypoid lesions in the ureter distal to ureteral stones were included during ureteroscopy. Computed tomography was performed on all enrolled patients three months after the procedure. Follow-up ureteroscopy was performed only if the patient consented, due to the need for general anesthesia and ethical considerations.

**Results:**

Among the 35 patients who were followed up, 14 had fibroepithelial polyps and 21 had inflammatory polyps. Twenty of the followed-up patients underwent ureteroscopy, and nine of them had fibroepithelial polyps. Although fibroepithelial polyps did not disappear in the follow-up ureteroscopy (p = 0.002), the rate of postoperative hydronephrosis was not higher in the fibroepithelial group than in the inflammatory group. Postoperative ureteral stricture and moderate-to-severe hydronephrosis were found to be closely related to the number of resected polyps, regardless of the type of polyp (p = 0.014 and 0.006, respectively).

**Conclusion:**

Fibroepithelial polyps in the ureter may persist after treatment of adjacent ureter stones. However, conservative management may be preferable to active removal of ureteral polyps because fibroepithelial polyps may not contribute to clinically significant hydronephrosis after surgery, and inflammatory polyps disappear spontaneously. Hasty resections of polyps may increase the risk of ureteral stricture.

**Supplementary Information:**

The online version contains supplementary material available at 10.1186/s12894-023-01249-y.

## Introduction

**Fig. 1:**
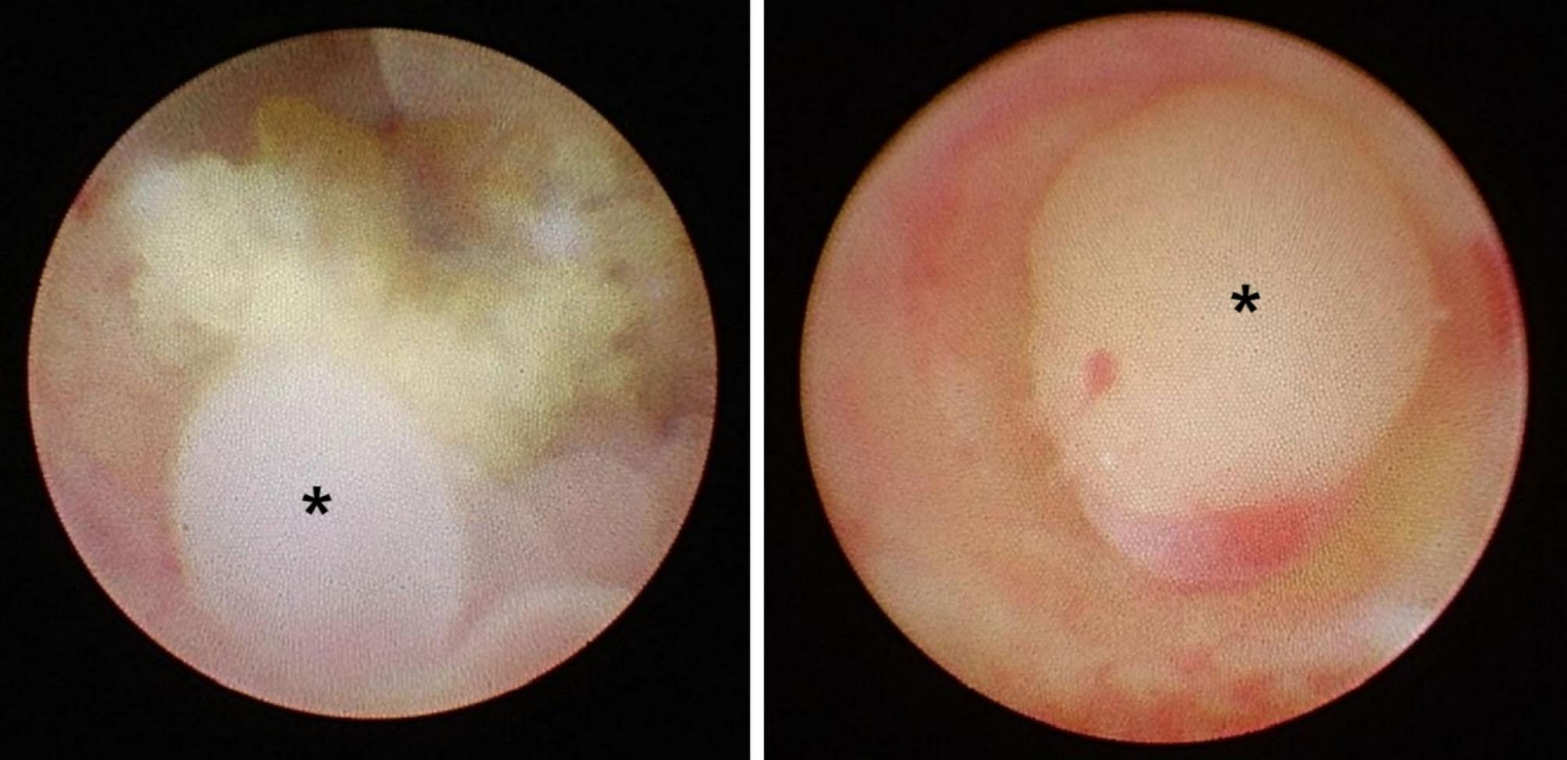
Ureteroscopic view of polyps Rough surfaced ureter stone was impacted and reactive polypoid lesions were identified (left). A fibroepithelial polyp was shown in the center of ureter (right). Asterisks (*) indicate polypoid lesions

Ureteral stones can disturb the urinary drainage and result in hydronephrosis, which causes renal colic. Reactive inflammation of ureteral mucosa around the stone will develop when the stone resides at the ureter without distal migration, thus leading to edematous and polypoid mucosal tissues. In the case of impacted stones, chronic stone irritation to the adjacent mucosa would be accelerated under the hydrostatic pressure of the renal pelvis. The edematous and polypoid lesion would be expected to disappear after discontinuation of stone irritation. However, in some cases, polypoid lesions around impacted stones cannot be discriminated with a fibroepithelial polyp (FEP), a real polyp (Fig. 1).

The etiology of a fibroepithelial polyp has yet to be established. Obstruction, infection, trauma, chronic irritation, hormonal imbalance, and developmental defects have been mentioned as possible causes [1]. In a recent systematic literature review, chronic irritation by urolithiasis has again been highlighted as a main etiology of the FEP [2]. However, it has been reported that the FEP could be found even in a young age group without urolithiasis [3]. Thus, the mechanism of FEP development remains unclear. Li et al. [4] have reviewed 37 reports out of a total of 126 cases of FEP with hydronephrosis in children and suggested endoscopic treatment for one or two pedunculated polyps and pyeloplasty in the case of multiple polyps. However, these suggestions could be valid in the case of non-calculus hydronephrosis and a positive filling defect in children.

As a rare cause, especially in the endemic area, ureteral involvement of schistosomiasis can create ureteral polypoid lesions where chronic irritation of mucosa by ova may lead to precancerous conditions such as squamous metaplasia [5]. In addition, Urothelial tumors such as transitional cell carcinoma mimic the benign FEP, establishing the correct preoperative diagnosis difficult [6]. These reasons may enforce surgeons to perform biopsy for the polypoid lesions during ureteroscopy.

When polypoid lesions are detected by chance during ureteroscopy for ureteral stone removal, surgeons may expect that such lesions would disappear or at least regress after stone surgery. Surgeons may need to make a decision as to whether they should remove all polyps simultaneously with stone retrieval or leave the polyps as they are only with a biopsy. However, the natural course of a stone-related polypoid lesion remains unclear, particularly after stone treatment. Surgeons are less likely to immediately perform invasive treatments such as ureteroureterostomy or pyeloplasty while encountering a ureteral polyp during ureteroscopic stone treatment. Therefore, this study aimed to observe the natural course of ureteral polyps associated with ureter stone treatment. It is expected that this study can serve as a resource for endourologists to refer to when they to encounter a polypoid lesion during ureteroscopy for stone removal.

## Methods and materials

### Ethics

This prospective observational study was conducted at six teaching hospitals with 800 to 1,800 beds between August 2019 and July 2021. In the present study, a surgeon in each hospital who specialized in urolithiasis with more than 5 years and 500 experiences of ureteroscopy conducted each of the procedures. A central ethics committee first approved the present study in the Catholic Medical Centre, The Catholic University of Korea College of Medicine, Seoul, South Korea. (Approval no. VC19OEDI0185). The respective local ethics committees then approved it. The local ethics committees allowed study collaborators to gather and access patient data and output all data in case report forms at each institute. When surgeons at these institutions completed case report forms, they electronically and personally sent these data to the main director. Informed written consent was obtained from all individual participants included in this prospective observational study.

### Study protocol

The inclusion criteria were: (1) patients over 20 years old and (2) patients having obvious polypoid lesions endoscopically obstructing the ureter distal to a ureteral stone during ureteroscopic ureteral stone treatment. In most cases, it was recommended that only a part of one or two polypoid lesions among multiple polypoid lesions be biopsied for pathological confirmation. However, surgeons could choose to remove all polypoid lesions if there were a few lesions during ureteroscopy. Although there were slight variations, a consensus of energy setting of Ho:YAG laser in the present study for resection of polyps converged at 0.8 ~ 1.2 J and 5 ~ 10 Hz. It has been suggested that over 2.0 J and/or over 20 Hz setting can raise the temperature of ureteral cavity even in a couple of seconds, which makes ureteral mucosa be vulnerable to the stricture [7]. In all cases, selective urinary cytology was checked during ureteroscopy. A ureteral stent was placed for 1 ~ 2 weeks after stone treatment. The exclusion criteria were: (1) patients who did not want to be enrolled or withdrew from the study enrollment, (2) patients having a single kidney, or (3) patients exhibiting malignancy on the biopsy report. We showed intra-operative snapshots to each patient during enrollment, including polypoid lesions and stones.

Computed tomography (CT) was performed for all enrolled patients at three months postoperatively to check for the presence of hydronephrosis. The postoperative CT scan was performed without contrast enhancement because the biopsy results had been already obtained, and there could be ethical issues including radiation exposure as well as contrast material toxicity. Grading of hydronephrosis was suggested by the Society for Fetal Urology using ultrasound [8]. Since there is no separate grading system for adults and the classification is based on changes in the appearance of hydronephrosis, we have determined that it can also be used for adults. Thus, we defined moderate hydronephrosis when the evidence of dilated minor calyx existed in a CT scan and severe hydronephrosis when the renal parenchyma was significantly thinned. Follow-up ureteroscopy was recommended when patients desired to know whether or not the polypoid lesions would disappear. Regardless of patients’ willingness, surgeons strongly recommended follow-up ureteroscopy for cases with moderate-to-severe hydronephrosis in postoperative CT scans. We tried to remove ureter stones using semi-rigid ureteroscope initially. In cases of stone migration into renal pelvis or difficult cases for approaching to the lesions, flexible ureteroscope (LithoVue™, Boston Scientific, Marlborough, MA, US) was immediately introduced. However, flexible ureteroscope was eventually put to use to navigate the upper ureter, renal pelvis, and calyces to confirm whether there is another pathological lesion or not. Furthermore, it was inevitable to use the flexible ureteroscope at three months postoperatively because we should keep the consistency to evaluate the presence of postoperative stricture endoscopically. We defined ureteral stricture under ureteroscopic vision when the tip of the flexible ureteroscope (7.7Fr) could not pass through the previous operative area as it was suggested by Ulvik et al. [9].

**Fig. 2:**
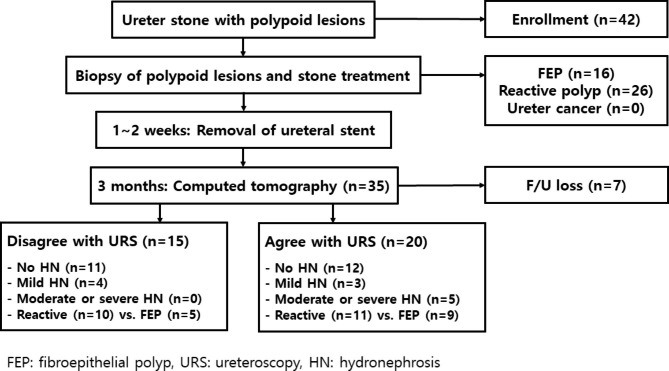
Flow chart of the present study Among 42 enrolled patients, 35 patients completed the present study, of which 20 patients agreed to a follow-up ureteroscopy

When mild hydronephrosis was found in CT scans and the patient did not want to undergo any invasive procedure of ureteroscopy, diuretic mercaptoacetyltriglycine renal scan was performed to evaluate the ureteral patency. We decided to observe the patients without further treatment if the T1/2 of mercaptoacetyltriglycine renal scan was lower than 20 min [10] (Fig. 2).

### Statistics

Mann-Whitney’s U test was used to compare the group with FEP to that of reactive polyps. The Chi-square test or Fisher’s exact test was used to analyze the binominal relationship between the two groups. All statistical analyses were performed using the statistical package IBM-SPSS for Windows Version 23.0 (SPSS Inc., Chicago, IL, USA).

## Results

In total, 42 patients were enrolled in the present study. The mean ± standard deviation of ages (year), body mass indexes (kg/m^2^), Hounsfield Units (HU), and longest diameters (mm) of the stones were 57.57 ± 10.73, 25.37 ± 3.19, 1138.74 ± 349.39, and 11.10 ± 4.05, respectively. FEPs were found in 16 cases, and abnormal urinary cytology was not found in any case. The baseline characteristics are described in Table 1.

**Table 1:** Patients’ baseline characteristics

Polyp type	Total (n = 42)	FEP (n = 16)	Reactive (n = 26)	p-value
Age (year)^†^	57.57 ± 10.73	57.13 ± 11.30	57.75 ± 10.58	0.835
Sex (Male)	34/42	15/16	19/26	0.102
BMI (kg/m^2^)^†^	25.37 ± 3.19	25.17 ± 2.73	25.49 ± 3.49	0.897
HTN^‡^	24/42	10/16	14/26	0.411
DM	9/42	4/16	5/26	0.471
Gout	0/42	0/16	0/26	NA
Hypercalcemia	3/42	0/16	3/26	NA
Laterality (Right)^‡^	24/42	11/16	13/26	0.192
Level of ureter				0.951
Upper	32	12	20	
Middle	3	1	2	
Lower	7	3	4	
HU^†^	1138.74 ± 349.39	1200.19 ± 321.78	1100.92 ± 366.29	0.534
Stone size (mm)^†^	11.10 ± 4.05	12.47 ± 4.30	10.26 ± 3.73	0.150
Stone surface^‡^				
Rough or spiculated	28/42	10/16	18/26	0.452
Hydronephrosis^‡,*^				
Moderate to severe	19/42	8/16	11/26	0.433
Number of Polyps				
5 or more	15/42	7/16	8/26	0.511
F/U CT	35/42	14/16	21/26	0.454
F/U URS^‡^	20/42	9/16	11/26	0.365
Stone analysis				
Calcium Oxalate	41/42	15/16	26/26	NA
Calcium Phosphate	3/42	1/16	2/26	NA
Uric acid	1/42	1/16	0/26	NA
Struvite (mixed) ^‡^	15/42	6/16	9/26	0.452
Others	0/42	0/16	0/26	NA

^†^: *p*-values were measured using Mann-Whitney test

^‡^: *p*-values were measured using Chi-squared test

*: There was no patient without pre-operative hydronephrosis

The other *p*-values were measured using Fisher exact test

FEP: fibroepithelial polyp, BMI: body mass index, HTN: hypertension, DM: diabetes mellitus, HU: Hounsfield unit, CT: computed tomography, URS: ureteroscopy,

There were no significant differences in the baseline clinical parameters between the groups of FEP and the reactive polyps. In 15 (35.7%) patients, the stones were surrounded by five or more polyps among all cases. However, in terms of size, there were no giant polyps. Thirty-five patients completed the study by undergoing CT scans at three months after the operation (Table 2). Postoperative hydronephrosis was found in 12 cases among these patients, with five cases having moderate-to-severe hydronephrosis. The occurrence rates of postoperative hydronephrosis did not significantly differ between the two groups.

**Table 2:** Outcomes at three months after operation

Polyp type	Total	FEP	Reactive	*p*-value
CT follow-up	35	14	21	
Hydronephrosis	12	3	9	0.282
Mild	7	2	5	0.676
Moderate to severe	5	1	4	0.627
URS follow-up	20	9	11	
Persistent polyp	6	6†	0	0.002
Stricture	5	1‡	4	0.319

All cases with moderate to hydronephrosis showed obvious stricture under the vision of URS.

†: All polyps disappeared in three cases of FEP group where all the polyps had been removed during previous URSs. In the other six cases of FEP group, a part of one to two polyps was biopsied and most of the polyps had been preserved

‡: In a case with stricture in FEP group, more than five polyps had been removed using Holmium:Yag laser during previous URS.

*p*-values were measured using Fisher exact test

**Fig. 3:**
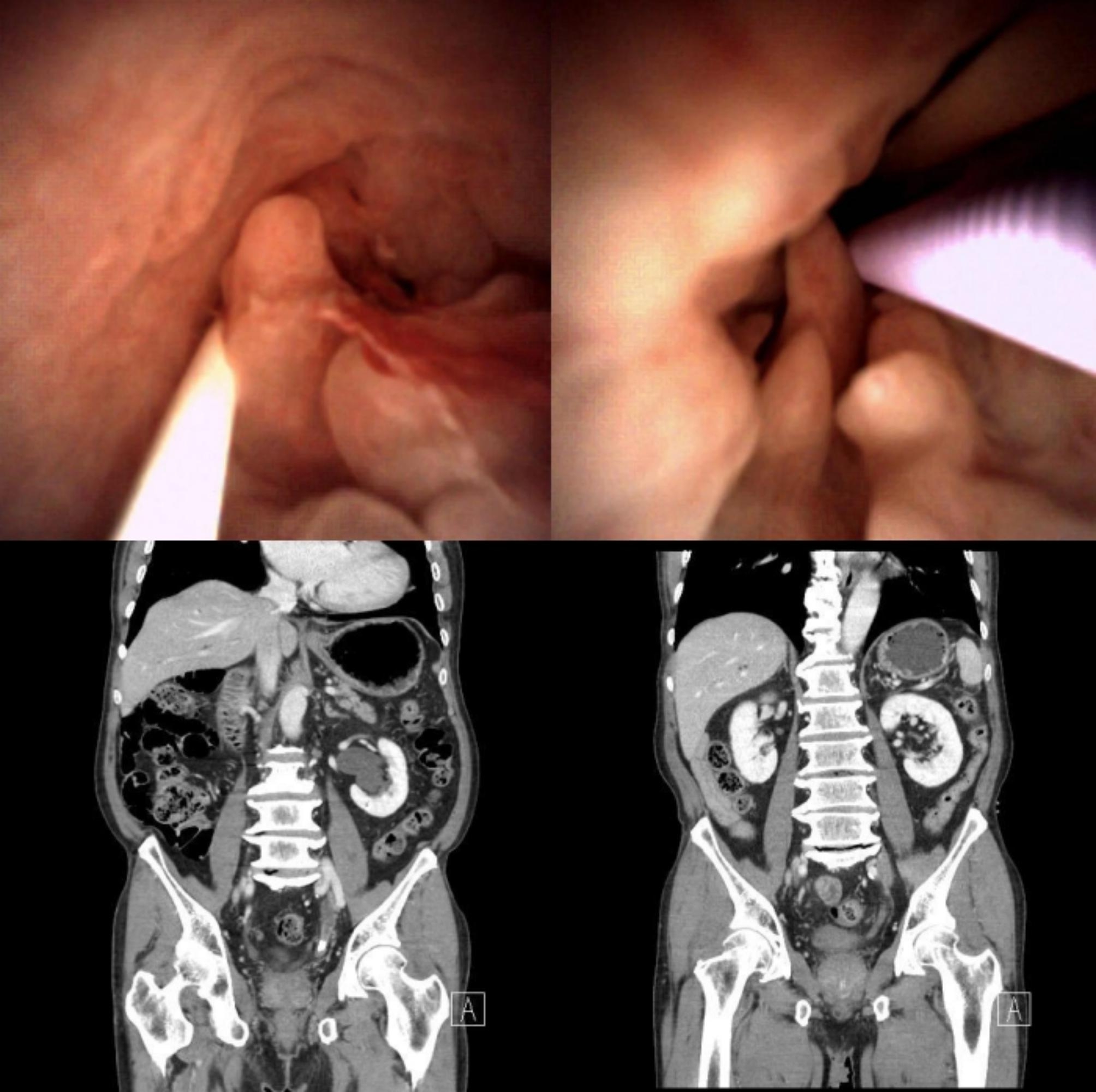
Fibroepithelial polyps with ureter stone Fibroepithelial polyp during ureter stone treatment (left top). Lt. lower ureter stone in preoperative computed tomography (left bottom). Persistent fibroepithelial polyp during follow-up ureteroscopy (Right top). No more hydronephrosis in postoperative follow-up computed tomography (Right bottom)

FEP: fibroepithelial polyp, CT: computed tomography, URS: ureteroscopy,

Twenty (57.1%) of the followed-up patients underwent ureteroscopy. FEP did not disappear unless we had resected all polyps during the previous ureteroscopic surgery (Fig. 3; Table 2). Postoperative moderate-to-severe hydronephrosis was only found in a single case, which was attributed to stricture of the previous ureteroscopic polypectomy site in the FEP group. However, there were four cases of moderate-to-severe hydronephrosis in the group of the reactive polyps. Each case also showed ureteral stricture in follow-up ureteroscopy (Table 2), although there was no differences in the occurrence rates of postoperative hydronephrosis and ureteral stricture. Of the remaining fifteen patients who did not show any stricture in follow-up ureteroscopy, none of them showed moderate-to-severe hydronephrosis postoperatively. Four patients with mild hydronephrosis who had reactive polyps disagreed with follow-up ureteroscopy (Fig. 2). Instead, they underwent diuretic mercaptoacetyltriglycine renal scan where the mean ± standard deviation of T1/2 (min) was 8.95 ± 2.15 (Supplementary Fig. 1). The number of resected polyps (5 or more), rather than preoperative hydronephrosis, stone size, initial number of polyps, or pathology (FEP), was closely related to postoperative moderate-to-severe hydronephrosis and ureteral stricture (Table 3).

**Table 3:** Risk factor analysis for postoperative outcomes

	Follow-up Computed Tomography (n = 35)		*p*-value	Follow-up Ureteroscopy (n = 20)		*p*-value
	Moderate to Severe HN (n = 5)	Non- or Mild HN (n = 30)		Stricture (n = 5)	No Stricture (n = 15)	
Age (years)	66.0 ± 7.18	57.70 ± 10.63	0.086	66.0 ± 7.18	55.27 ± 8.49	**0.033**
BMI (kg/m^2^)	24.49 ± 4.29	25.20 ± 3.08	0.802	24.49 ± 4.29	25.02 ± 2.58	0.800
HU	1002.0 ± 412.27	1177.80 ± 280.26	0.448	1002.0 ± 412.27	1141.40 ± 274.85	0.612
Stone size (mm)	9.91 ± 3.97	11.79 ± 4.15	0.506	9.91 ± 3.97	12.98 ± 4.65	0.266
Sex (male)	3	28	0.089	3	15	0.053
Hypertension	4	18	0.630	4	7	0.319
Diabetes	0	7	0.559	0	5	0.266
FEP	1	13	0.627	1	8	0.319
Laterality (Right)	2	19	0.369	2	10	0.347
Rough stone surface	2	21	0.313	2	10	0.347
Initial hydronephrosis†	2	14	1.000	2	7	1.000
Number of polyp (≥ 5)	4	11	0.141	4	8	0.603
Resected polyp (≥ 5)	4	6	**0.006**	4	2	**0.014**
Struvite component	2	9	0.640	2	4	0.613

†: Moderate to severe hydronephrosis

*p*-values were measured using Mann-Whitney test or Fisher exact test

BMI: body mass index, HU: Hounsfield unit, FEP: fibroepithelial polyp

## Discussion

This study provides helpful information about the treatment strategy when endourologists encounter polypoid lesions during ureteroscopic surgery, because there has not been clear evidence regarding the follow-up results of those lesions. Ludwig et al. have suggested a three-month follow-up strategy by CT scans in a systematic review [2]. Despite the existence of a systematic review including 75 articles, follow-up data was only available for 57 out of 134 patients. Thus, collecting follow-up data of polypoid lesions associated with ureter stone is not an easy process in a single study due to the rare nature of relevant cases. Furthermore, follow-up ureteroscopy may be far more difficult because of a need for general anesthesia. Previous studies have typically shown follow-up results of ureteral polyps with the presence of hydronephrosis, particularly in pediatric patients [3–4]. Although some case reports showed stone-related ureteral polyps that were incidentally found in adult patients [11–14], they only provided limited information without detailed ureteroscopic findings of the ureteral polyps. The present study showed that FEP did not disappear unless the endourologists resected them. However, reactive polyps disappeared in all cases, although there was no significant difference in the rates of hydronephrosis in CT scans between the two groups. This finding may indicate that endourologists do not need to resect all polyps and that active removal of ureteral polyps is not mandatory. The ureteroscopic biopsy is only needed to rule out malignancy.

The occurrence rate of ureteral stricture is one of the essential factors when surgeons establish a follow-up plan for ureteral polyps. Xi et al. reported a stricture rate of 26.2% in patients with ureteral polyps [16], which is higher than that in the present study. This data was analyzed in a retrospective manner using intravenous urography, not using ureteroscopy. They resected the polyps with Ho:YAG laser of 10 to 15 Hz, although we could not obtain sufficient data on the laser energy setting. Several studies [12, 15] have shown similar rates of the ureteral stricture with Ho:YAG laser lithotripsy to ours. The contact technique on a polyp after ureteroscopic biopsy is not recommended for treating only ureteral stones, because high energy transmission to ureteral tissues can increase the risk of postoperative ureteral stricture and hydronephrosis. Although several reports have presented cases in which they had resected and coagulated the stalks of the polyps during ureteroscopy [14, 16], the number of resected polyps or the range of lasering was not precisely documented in those works. Therefore, we should be aware of the thermal effect of Holmium lasers on ureteral mucosa and the deep muscle layer. To this end, Dong et al. emphasized the prevention strategies for ureteral stricture following ureteroscopy. They supposed that the Holmium laser lithotripsy might have a higher incidence of postoperative ureteral stricture than the pneumatic lithotripsy [17]. The result was consistent with that obtained in the present study. Five cases of moderate-to-severe hydronephrosis, which in turn were revealed as ureteral stricture under the vision of ureteroscopy, were found to be closely related to the aggressive resection of five or more polyps (Tables 2 and 3).

Regarding the resection of FEP, Li et al. have also recommended that an endoscopic resection should be done in the case of a few, pedunculated polyps, thus implying the possibility of postoperative ureteral stricture after endoscopic resection for multiple polyps [4]. Recently, an interesting study has been published showing the efficacy and safety of endoscopic resection of FEP using thulium laser [13]. The authors conducted a retrospective multicenter study wherein 21 patients were followed up with using CT urography. After all polyps were removed, postoperative ureteral stricture did not occur [13]. However, a direct comparison between the previous study conducted by Gu et al. and ours was inappropriate for several reasons: (1) data were collected over ten years in a retrospective manner in that study, (2) the study was not associated with stone treatment, (3) the size and number of polyps were not described precisely, and (4) they used a different laser modality. In the majority of cases in the previous work, the polyps were not multiple. This might be one of the reasons why they did not experience postoperative ureteral stricture.

A hydronephrosis grading system was developed to establish an objective tool. Ultrasonographic classification from grade I to IV has been well set for evaluating antenatal hydronephrosis [18]. The same classification can be applied to the severity of hydronephrosis based on CT scans. Calyceal blunting and cortical thinning can be good criteria for differentiating moderate from mild or severe hydronephrosis [19], although the criteria can be subjective and differ between reviewers [20]. The authors of the present study attempted to check the mercaptoacetyltriglycine renal scan to prevent under-diagnosed mild hydronephrosis in cases in which relevant patients were not willing to have ureteroscopy.

Several case reports of FEP showed that moderate-to-severe hydronephrosis was an initial presenting sign. In the study mentioned above conducted by Gu et al. [13]., the preoperative moderate-to-severe hydronephrosis was seen in 15 (71.4%) of 21 patients without stone-related obstruction. It seemed that the moderate-to-severe hydronephrosis developed closely to the evaluation time in the previous study. If it was clinically significant, moderate-to-severe hydronephrosis had been sustained due to the presence of FEP for a long time, and the hydronephrosis was detected incidentally at a middle or older age, the ipsilateral renal function might already have deteriorated and might not be restored regardless of the resection of FEP [21]. Due to the lack of information regarding the relationship between the severity of hydronephrosis and the nature of FEP, we can only assume that some cases of FEP without stone-related obstruction might present a symptom of flank pain or a sign of hydronephrosis with relatively preserved renal function for the following reasons: it might gradually grow as a giant FEP, which could worsen the severity of hydronephrosis [22] or other complications like intussusception could accelerate the hydronephrosis [23]. However, based on the present study, which did not exhibit those complications, removal of the stone alone as a critical cause of hydronephrosis could offer satisfactory postoperative results while avoiding the use of additional invasive procedures (Fig. 3).

The present study has several limitations. First, each surgeon did not show the energy setting of the Ho:YAG laser when they handled the polyps. But, as aforementioned, a consensus of energy setting of Ho:YAG laser in the present study for resection of polyps converged at 0.8 ~ 1.2 J and 5 ~ 10 Hz. Second, we could only investigate a small number of FEP, as this is a rare type of benign tumor. Third, ureteroscopy was omitted in 15 (42.9%) patients who did not agree to undergo another instance of ureteroscopic surgery. Ethically, researchers could not impel the patients to have follow-up ureteroscopy without evidence of severe hydronephrosis, and we added a mercaptoacetyltriglycine renal scan to prevent under-diagnosed mild hydronephrosis. Fourth, the definition of ureteral stricture under the vision of ureteroscopy is somewhat arbitrary, although a similar method was applied in the previous research [9]. However, postoperatively moderate-to-severe hydronephrosis in a CT scan well reflected a stricture under the ureteroscopic view (Table 2). Lastly, it was difficult to accurately record the duration of stone impaction. That is because the symptom duration is largely dependent on the patient’s memory and many patients without any symptom were diagnosed during medical checkup.

## Conclusion

FEP may not disappear spontaneously after the removal of an adjacent stone whereas inflammatory, reactive polyps regresses after stone retrieval. Endourologists may not need to resect all polypoid lesions during ureteroscopic stone treatment, because polyps remaining after stone removal may not contribute to the postoperative hydronephrosis or stricture. Instead, the active removal of ureteral polyps using laser may lead to ureteral stricture.

## Electronic supplementary material

Below is the link to the electronic supplementary material.

Additional File 1: Diuretic renal scan in patients with postoperative mild hydronephrosis

## Data Availability

The datasets used during the current study available from the corresponding author on request.

## Abbreviations & Acronyms

CT: Computed tomography
FEP: fibroepithelial polyp
HU: Hounsfield Unit
