# Supplementary material for: The natural course of incidental ureteral polyp during ureteroscopic surgery: KSER research
Source: BMC Urol. 2023 Jun 3;23:101. doi: 10.1186/s12894-023-01249-y (PMC10268500; doi:10.1186/s12894-023-01249-y)

Supplementary files

Supplementary figure 1

Right upper ureter stone and moderate-to-severe hydronephrosis was identified (left and middle top). During ureteroscopy, polypoid lesions were found, which was revealed as reactive polyps (right top). Mild hydronephrosis was remained postoperatively (left bottom). In this case, the patient disagreed with follow-up ureteroscopy. T1/2 (min) of left and right were 4.33 and 8.0, respectively, in mercaptoacetyltriglycine renal scan (blue dotted line: left, purple bold line: right).

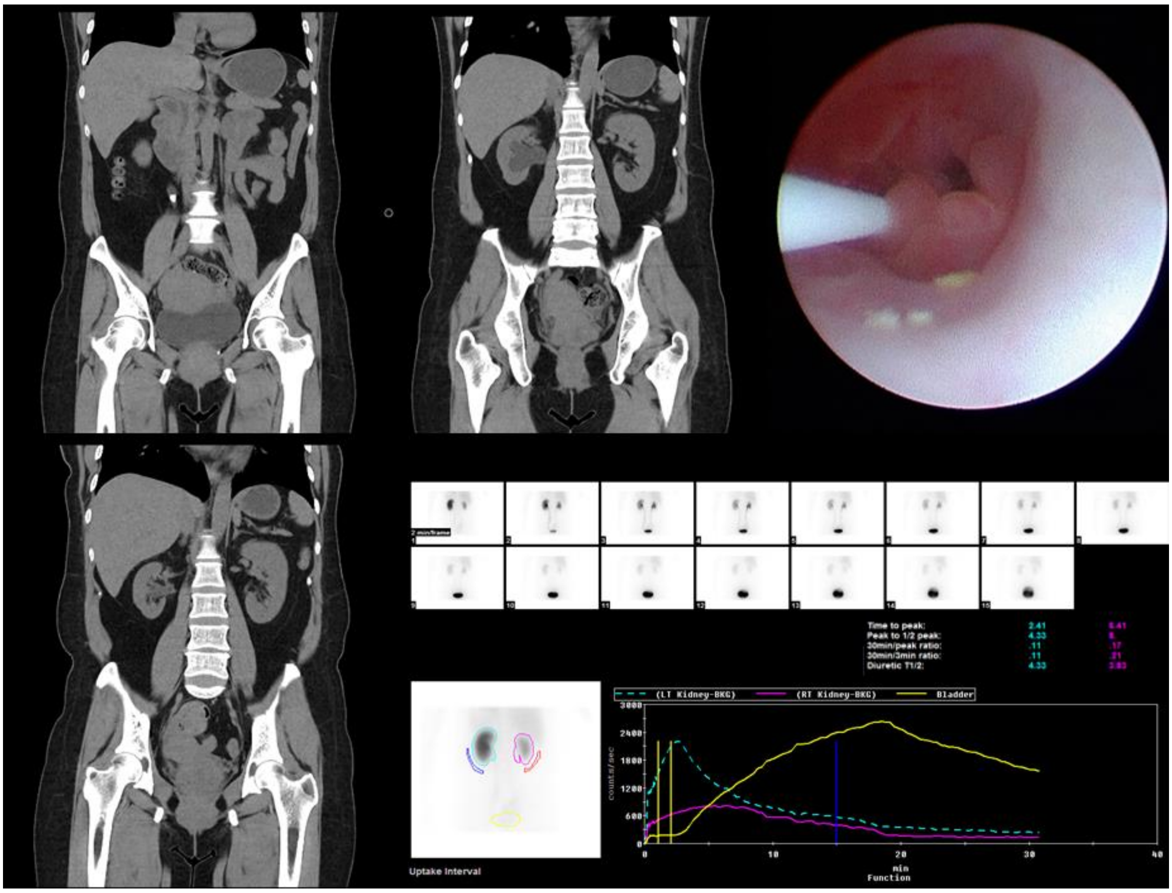

Supplement: Supplementary file 1 — Additional File 1: Diuretic renal scan in patients with postoperative mild hydronephrosis [file 12894_2023_1249_MOESM1_ESM.pdf]
